# CT-guided radiofrequency ablation of the extracranial cranial nerve for the treatment of Meige’s syndrome

**DOI:** 10.3389/fnins.2022.1013555

**Published:** 2022-10-06

**Authors:** Bing Huang, Xin-dan Du, Ming Yao, Hui-dan Lin, Wen-hua Yu, Qing-he Zhou

**Affiliations:** ^1^Department of Pain Medicine, Affiliated Hospital of Jiaxing University, Jiaxing, China; ^2^Department of Pain Medicine, Redcross Hospital of Hangzhou, Hangzhou, China; ^3^Department of Pain Medicine, First Hospital of Ningbo, Ningbo, China; ^4^Department of Neurosurgery, Affiliated Hangzhou First People’s Hospital, Zhejiang University School of Medicine, Hangzhou, China

**Keywords:** Meige’s syndrome, radiofrequency ablation, facial nerve, trigeminal nerve, CT

## Abstract

**Background:**

Meige’ s syndrome, a rare form of dystonia, lacks effective treatment. The purpose of this study was to determine the effects of CT-guided percutaneous extracranial radiofrequency ablation of the facial and/or trigeminal nerves in the treatment of Meige’s syndrome.

**Methods:**

A total of 10 patients were enrolled in this study, with the numbers of blepharospasm dystonia syndrome (BDS), oromandibular dystonia syndrome (ODS), and blepharospasm combined with oromandibular dystonia syndrome (B-ODS) being 7, 1, and 2, respectively. BDS patients underwent radiofrequency ablation of the bilateral stylomastoid foramen facial nerve; ODS patients underwent radiofrequency ablation of the bilateral foramen oval trigeminal mandibular branch, and B-ODS patients underwent radiofrequency ablation of the bilateral stylomastoid foramen facial nerve and foramen oval trigeminal mandibular branch. The therapeutic effects and complications were observed.

**Results:**

All 10 patients in this series experienced improved Meige’s syndrome-related symptoms after extracranial radiofrequency ablation of the cranial and/or mandibular branches of the extracranial trigeminal nerve. Adverse events included class II–III facial paralysis and/or mandibular skin numbness. Two patients had recurrences at the 18th and 22nd months postoperatively, respectively; the other patients were being followed up.

**Conclusion:**

These results shown that CT-guided radiofrequency ablation of bilateral stylomastoid foramen facial nerve and/or oval foramen trigeminal mandibular branch can effectively treat the corresponding types of Meige’s syndrome. According to preliminary observations, the therapeutic effect may last more than 18 months.

## Introduction

Meige’s syndrome, also known as segmental craniometrical dystonia, is a rare form of cranial dystonia characterized by blepharospasm (BDS) and oromandibular dystonia (ODS), as well as complex movement of the lower facial muscles, jaw, mouth, tongue, pharyngeal, and cervical muscles (Pandey and Sharma, 2017). BDS is the most common and incapacitating manifestation in a patient with Meige’s syndrome, which begins unilaterally in nearly 25% of patients and quickly progresses to bilateral (Tolosa and Klawans, 1979), with progressive worsening of BDS and spreading of symptoms involving the oromandibular, cervical, and limb muscles (Weiss et al., 2006; Abbruzzese et al., 2008). Meige’s syndrome is classified into three subtypes based on the distinct craniometrical muscles involved: BDS, ODS, and blepharospasm combined with oromandibular dystonia (B-ODS) (Marsden, 1976).

Oral medications (LeDoux, 2009) and botulinum toxin injections (Bhidayasiri et al., 2006; Czyz et al., 2013), are currently used as therapeutic options but have not yet achieved the required level of clinical resolution. Although deep brain stimulation has emerged as an alternative treatment option for intractable patients (Markaki et al., 2010; Reese et al., 2011), the treatment necessitates excessive technical requirements and the cost is high.

Our previous studies established that CT-guided percutaneous stylomastoid foramen puncture and radiofrequency ablation are effective for treating hemifacial spasm (Huang et al., 2021),and that CT-guided percutaneous foramen oval puncture and radiofrequency ablation are effective for treating mandibular branch of trigeminal neuralgia and refractory V3 trigeminal neuralgia (Huang et al., 2019a; Lin et al., 2021). Based on these prior studies, here we report our experience with 10 patients who underwent CT-guided extracranial radiofrequency ablation of the cranial nerve for the treatment of Meige’s syndrome.

## Materials and methods

This study was approved by the Ethics Committee of the Affiliated Hospital of Jiaxing University, Jiaxing, China (LS2019-013) on 20 May 2019. A total of 10 patients diagnosed with Meige’s syndrome were enrolled from April 2019. All study subjects volunteered to participate in this prospective, observational study and provided consent to use their health data and portraits for future publications.

### Inclusion and exclusion criteria

This study enrolled patients with Meige’s syndrome. Before surgery, all individuals consented to a cranial CT examination to rule out cerebral space-occupying lesions. All patients underwent a three-dimensional time of flight magnetic resonance angiography (3D-TOF MRA) and there was no concomitant vascular compression of bilateral facial nerves. Synchronous contraction of the bilateral orbicularis oculi muscle and explosive discharge of the bilateral orbicularis oris muscle were observed on facial electromyography before the operation, and no abnormal muscle response was noted. None of the subjects had a history of Parkinson’s disease or tremor, long-term history of taking psychosedative drugs, craniocerebral trauma, cerebrovascular accident, or encephalitis. None of the patients had any history of deep brain stimulation. Contraindications to radiofrequency ablation treatment such as coagulation dysfunction, infection of the puncture site, and cardiac pacemaker were also excluded.

### Treatment options

Patients with BDS were treated with radiofrequency ablation of bilateral stylomastoid foramen facial nerve under CT guidance; patients with ODS were treated with radiofrequency ablation of bilateral foramen oval trigeminal mandibular branch under CT guidance, and patients with B-ODS were treated with radiofrequency ablation of bilateral stylomastoid foramen facial nerve and foramen oval trigeminal mandibular branch under CT guidance.

### Treatment of blepharospasm dystonia syndrome

**FIGURE 1:**
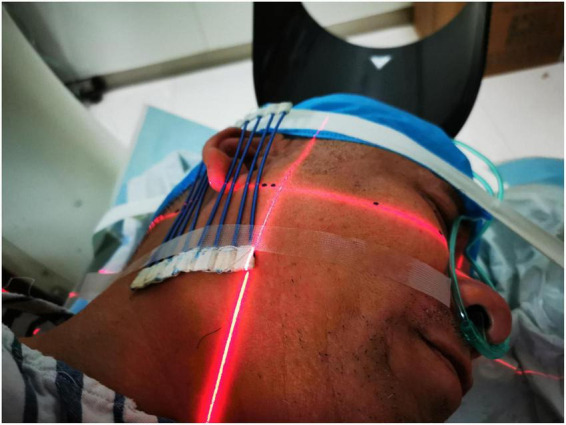
Placement of the CT positioning grids. CT positioning grids were placed anterior and posterior to the patient’s ear.

Before the procedure, study participants fasted for 4–6 h. After entering the CT examination room, the subject was placed in the lateral decubitus position with the head supported on a pillow. Upper limb venous access was established, and standard ASA monitors were implemented. Following confirmation of the puncture site, CT positioning grids were placed anterior and posterior to the patient’s ear (Figure 1). The mastoid region was scanned in 3-mm layers using a paranasal sinus CT scanning protocol. The stylomastoid foramen was identified on an image sequence [Syngo Multimodality Workplace (MMWP); Siemens] and set as the puncture target.

**FIGURE 2:**
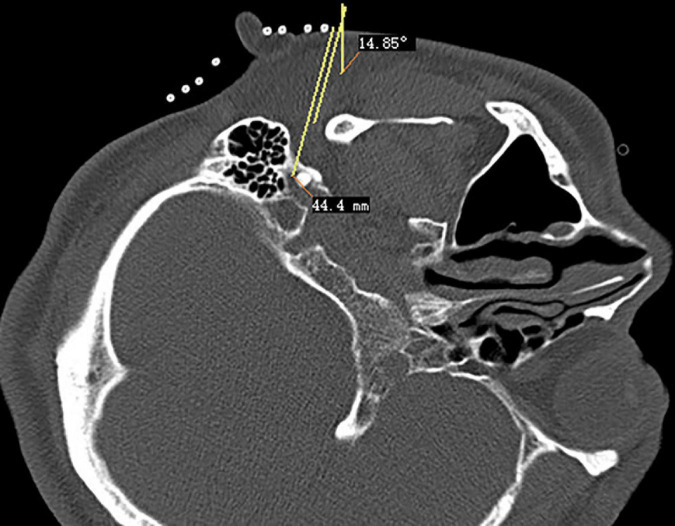
Puncture path design of stylomastoid foramen.

**FIGURE 3:**
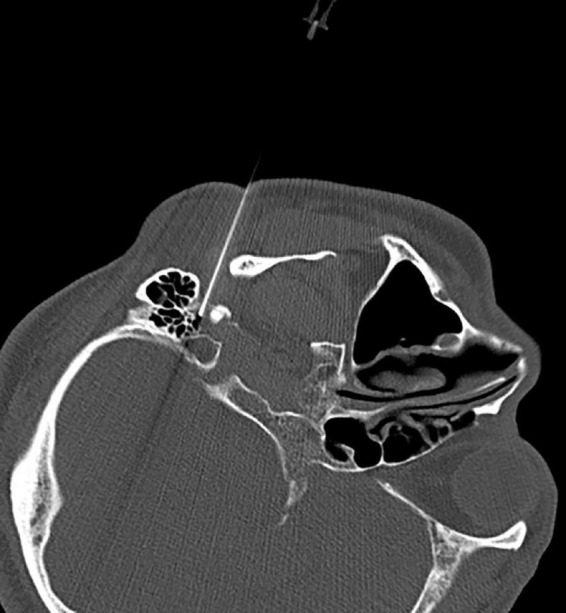
Puncturing of the stylomastoid foramen under intermittent CT guidance.

**FIGURE 4:**
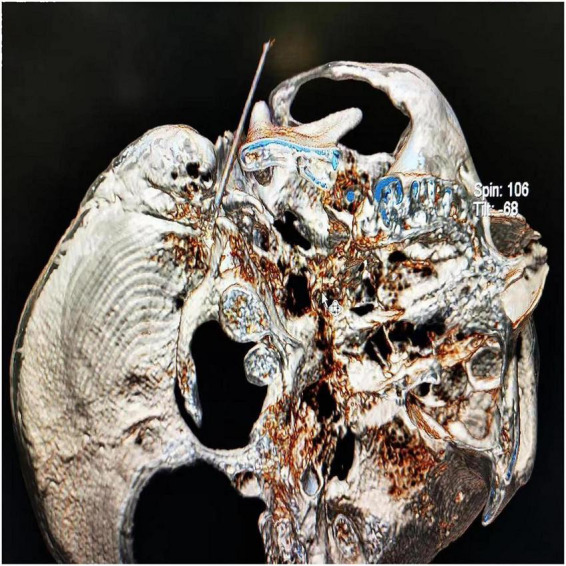
Three-dimensional reconstruction of the CT scans confirmed successful puncture of the stylomastoid foramen.

The CT layer with the stylomastoid foramen and without the bone barrier of the tympanic part of the temporal bone was selected as the puncture layer, and the CT measurement tool software was used to pull a straight line forward from the stylomastoid foramen. The intersection of the line and the skin is the puncture point (Figure 2). We determined the puncture depth (the distance from the puncture point to the target) and puncture angle (the angle between the puncture route and sagittal plane). After administering local anesthesia to the puncture site, a stylet 7-gauge radiofrequency needle with a length of 10 cm and an exposed end of 5 mm (Model 240100; Innomed Medical Technology Co., Ltd.) was gradually advanced toward the target under the guidance of intermittent CT inspection (Figure 3) and confirmed by three-dimensional reconstruction of CT scanning (Figure 4). The facial nerve was then stimulated with a radiofrequency probe at a frequency of 2 Hz and a current capacity of 0.5 mA (RF instrument model PMG230; Baylis Medical Co., Inc.). The positive facial muscle twitches from the stimulation indicated that the facial nerve was in close proximity (Supplementary Video 1).

Patients were required to puff their cheeks and close their eyes while receiving continuous radiofrequency ablation at a preset temperature of 65°C for 30 s. Whether air leakage occurred when the patient’s cheeks were bulging and the treatment side eye could be tightly closed during the treatment was closely observed. When the individual struggled to keep their eyes closed or their cheeks bulged, the ablation protocol was stopped, and the procedure was completed. After 30 s of radiofrequency ablation, if no air leakage occurred when puffing the cheeks and the eyes were tightly closed, there was an increase in the radiofrequency temperature by 5°C before the next cycle. Thus, a 5°C step heating radiofrequency was implemented until air leakage occurred on the treatment side during puffy cheeks and the eyes could not be tightly closed. Sedation was performed during the needle insertion process. The same method was used for contralateral treatment.

### Treatment of oromandibular dystonia syndrome

The patient was positioned supine on the CT scaffold, with thin pillows beneath the shoulders to keep the head backward by 15–25°. The patient’s head was fixed on the head frame of the CT table using a wide tape, and a CT positioning grid was placed on the lateral corner of the mouth of the affected side. The head positioning image was taken in the paranasal sinus mode, and the zygomatic face was scanned in the semi-coronal position with a 3 mm layer thickness. The scanning baseline was parallel to the line from the external earhole to the item point of the second molar, and the upper edge of the scanning frame reached the upper edge of the zygomatic arch. The scanned CT image was replayed, the layer containing foramen oval (puncture targets) on both sides selected as the puncture layer, the puncture path on this layer was designed by pulling a straight line from the target close to the inner edge of the coronal process to the facial soft tissue. The intersection of the straight line and skin was the puncture point. The distance from the target point to the puncture point (puncture depth) was measured, and the angle between the puncture direction and the sagittal plane (puncture angle) was determined using CT software.

Following routine disinfection and towel laying, local anesthesia at the puncture point was performed. A stylet 7-gauge radiofrequency needle with a length of 10 cm and an exposed end 5 mm close to the inner edge of the coronal process was inserted into the foramen oval along the designed puncture path, and the needle feeding was stopped when the needle tip was flat at the inner opening of the foramen oval to avoid the needle tip entering the brain.

If stimulation with high frequency (50 Hz) current (0.5 mA) or voltage (0.3 V) could induce alloesthesia in the innervation area of the trigeminal mandibular branch (lower lip, mandible, ototemporal area), and stimulation with low a frequency (2 Hz) current (0.5–1 MA) or voltage (0.3–0.5 V) could induce rhythmic jitter of the mandible (Supplementary Video 2), then standard radiofrequency thermocoagulation at 95°C was administered for 180 s (Lin et al., 2021).

**FIGURE 5:**
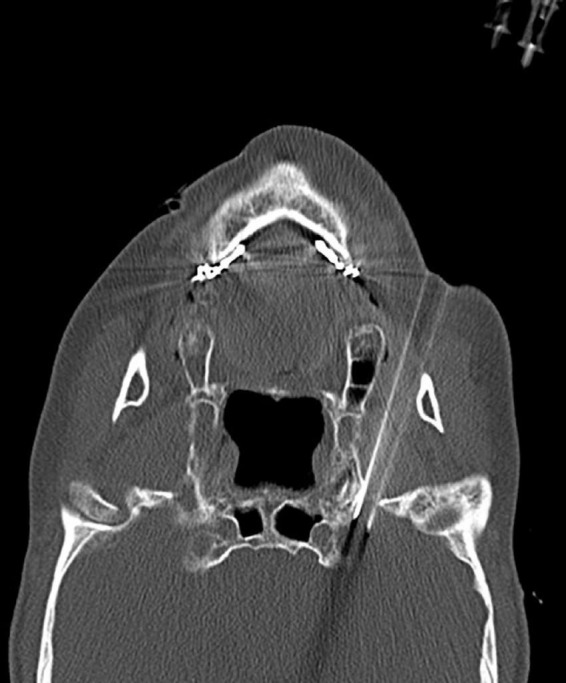
Two puncture needles are inserted into the foramen ovale.

The assumption was that CT scanning revealed the horizontal transverse diameter of the foramen oval on the affected side to be greater than 6 mm. In that case, double-needle bipolar RF ablation technology was used (Huang et al., 2019a): Two parallel puncture paths were designed with the inner and outer sides of the foramen oval as targets, respectively, and an RF needle was punctured into the inner and outer sides of the oval foramen along the designed path to the internal opening of the oval foramen. Double-needle bipolar radiofrequency ablation was performed after satisfactory electrophysiological testing (Figure 5).

### Treatment of blepharospasm combined with oromandibular dystonia syndrome

After the radiofrequency ablation treatment of the facial nerve *via* the stylomastoid foramen was conducted under CT guidance, radiofrequency ablation of the left and right mandibular branches of the extracranial trigeminal nerve *via* the oval foramen was performed using the methods described above, respectively.

### Following up and adverse events

All patients were followed up postoperatively to observe the therapeutic effects, and complications such as facial paralysis and skin numbness of the mandible were also observed. The definition facial paralysis was based on the report of House and Brackmann (1985).

## Results

### General characteristics

Ten patients (two men and eight women) were enrolled in this study. The average age was 57.6 ± 6.6 years, the average course of the disease was 3.1 ± 1.1 years, and the average follow-up time was 8.3 ± 10.5 months. The BDS, ODS, and B-ODS numbers were seven, one, and two, respectively.

### Effects and complications

All 10 patients in this series experienced improved Meige’s syndrome -related symptoms after extracranial radiofrequency ablation of a cranial nerve or/and mandibular branches of the extracranial trigeminal nerve. Two patients had recurrences at the 18th and 22nd months, respectively; the remaining eight patients were followed up for 1–9 months, respectively, and no recurrence was found. The adverse events included facial paralysis class II-III and/or mandibular skin numbness, and these complications improved over time (see Tables 1, 2).

**TABLE 1:** Characteristics and treatments of study patients.

Pt.	Diagnosis and relevant abnormal symptoms	Sex/age (years)	Duration of disease (years)	Previous treatment	Present treatment
1	BDS (blepharospasm)	Female/62	5	Botulinum toxin injection	Bilateral nerve radiofrequency ablation with 85°C for 30 s
2	BDS (blepharospasm)	Female/66	2	Botulinum toxin injection	Bilateral nerve radiofrequency ablation with 70°C for 23s
3	B-ODS (blepharospasm combined with oromandibular dystonia)	Female/60	3	Botulinum toxin injection	Bilateral nerve radiofrequency ablation with 65°C for 27 s combined with mandibular branch of bilateral trigeminal nerve radiofrequency ablation with 95°C for 180 s
4	BDS (blepharospasm)	Female/57	2	None	Bilateral nerve radiofrequency ablation with 65°C for 30 s
5	B-ODS (blepharospasm combined with oromandibular dystonia)	Male/54	4	Botulinum toxin injection	Bilateral nerve radiofrequency ablation with 90°C for 25 s combined with mandibular branch of bilateral trigeminal nerve radiofrequency ablation with 95°C for 180 s
6	BDS (blepharospasm)	Female/64	2	Botulinum toxin injection	Bilateral nerve radiofrequency ablation with 85°C for 20 s
7	ODS (oromandibular dystonia)	Male/43	3	None	Mandibular branch of bilateral trigeminal nerve radiofrequency ablation with 95°C for180 s
8	BDS (blepharospasm)	Female/58	4	None	Bilateral nerve radiofrequency ablation with 70°C for 30 s
9	BDS (blepharospasm)	Female/53	4	Botulinum toxin injection	Bilateral nerve radiofrequency ablation with 95°C for 30 s
10	BDS (blepharospasm)	Female/59	2	None	Bilateral nerve radiofrequency ablation with 80°C for 23 s

BDS, blepharospasm dystonia syndrome; ODS, oromandibular dystonia syndrome; B-ODS, blepharospasm combined with oromandibular dystonia syndrome.

**TABLE 2:** The outcomes and adverse events of radiofrequency ablation for Meige’s syndrome.

Pt.	Diagnosis and relevant abnormal symptoms	Follow up time	Treatment effect and recurrence	Adverse events	Adverse events lasted
1	BDS (blepharospasm)	31 months	Recurrence at the 18th month	Facial paralysis class II	Disappeared 2 months later
2	BDS (blepharospasm)	24 months	Recurrence at the 22th month	Facial paralysis class III	Disappeared 3 months later
3	B-ODS (blepharospasm combined with oromandibular dystonia)	9 months	No recurrence	Facial paralysis class III	Disappeared 3 months later; skin numbness of mandibular, lasted for 9 months
4	BDS (blepharospasm)	4 months	No recurrence	Facial paralysis class II	Disappeared 2 months later
5	B-ODS (blepharospasm combined with oromandibular dystonia)	4 months	No recurrence	Facial paralysis class II	Disappeared 3 months later; skin numbness of mandibular; lasted to now
6	BDS (blepharospasm)	3 months	No recurrence	Facial paralysis class III	Disappeared 2 months later
7	ODS (oromandibular dystonia)	3 months	No recurrence	Skin numbness of mandibular,	Numbness lasted to now
8	BDS (blepharospasm)	2 months	No recurrence	Facial paralysis class III, restored to class II	Lasted to now
9	BDS (blepharospasm)	2 months	No recurrence	Facial paralysis class II	Lasted to now
10	BDS (blepharospasm)	1 months	No recurrence	Facial paralysis class III	Lasted to now

BDS, blepharospasm dystonia syndrome; ODS, oromandibular dystonia syndrome; B-ODS, blepharospasm combined with oromandibular dystonia syndrome.

## Discussion

Meige’s syndrome is characterized by autonomic motor control dysfunction, basal ganglia thalamus cortical circuit interaction disorder, basal ganglia, and thalamus neurotransmitter imbalance, resulting in excitatory and inhibitory imbalance (LeDoux, 2009; Ribot et al., 2019; Ma et al., 2021). Jochim et al. (2020) reported that treating Meige’s syndrome with botulinum toxin A is safe and effective, lasting for only 2–4 months, and only a few patients experience toxic reactions (Hassell and Charles, 2020). Deep brain stimulation can be used to treat Meige’s syndrome by placing stimulation electrodes into the bilateral medial globus pallidus or the subthalamic nucleus. Nonetheless, only 50–70% of patients receive effective treatment. This technology requires stereotactic neuronavigation, which has stringent technical requirements and is too expensive for the majority of patients (Horisawa et al., 2018; Liu et al., 2021).

Dystonia is treated by blocking the generation and transmission of abnormal motor signals. Deep electrical stimulation is used to treat Meige’s syndrome because it blocks or interferes with the generation of abnormal movement signals of the hypothalamic nuclei, which is equivalent to treating the pacing system (signal source). Local injection of botulinum toxin blocks the transmission of abnormal motor signals at the neuromuscular junction, which is equivalent to the treatment of effectors. Radiofrequency ablation of the cranial nerve blocks the transmission of abnormal motor signals in the cranial nerve, which is equivalent to the treatment of the intermediate links of the conduction system. Different types of Meige’s syndrome affect different cranial nerves.

The facial nerve serves as the conduction system for BDS, whereas the mandibular branch of the trigeminal nerve serves as the main conduction system for ODS. The conduction system of BDS combined with ODS type involves the mandibular branch of the facial nerve and trigeminal nerve, involving pharyngeal muscle, lingual muscle, and neck muscle. The conduction system comprises glossopharyngeal, hypoglossal, and accessory nerves. Therefore, partial radiofrequency ablation of the above cranial nerves can theoretically block the transmission of abnormal motion signals to effectors (orbicularis oculi muscle, orbicularis oris muscle, and/or masticatory muscle, pharyngeal muscle, tongue muscle, sternocleidomastoid muscle, etc.), and thus alleviate Meige’s symptoms.

Our research group conducted an in-depth study on the treatment of trigeminal neuralgia using extracranial non-Gassel ganglion radiofrequency and discovered that radiofrequency therapy directed at the extracranial trigeminal nerve trunk can also achieve a satisfactory therapeutic effect (Huang et al., 2014, 2019b; Chen et al., 2019). Additionally, while using extracranial radio frequency, the puncture needle does not need to penetrate the skull to locate the semilunar ganglion. Nonetheless, the radio frequency for the mandibular branch can be realized at the foramen ovale (Huang et al., 2019a; Lin et al., 2021). Complications such as intracranial infection and intracranial hemorrhage associated with puncturing the semilunar segment of the intracranial trigeminal nerve can be avoided entirely, significantly improving the safety of radiofrequency treatment. Additionally, for the radiofrequency treatment of the facial nerve, we developed extracranial radio frequency technology, “CT-guided percutaneous puncture of the facial nerve in stylomastoid foramen for the treatment of facial spasm,” and successfully applied it in the clinic (Huang et al., 2021).

According to different syndrome types, this group adopted different cranial nerves for extracranial radiofrequency treatment: for BDS, which involves only the orbicularis oculi and orbicularis oris muscles innervated by the facial nerve, but not the masticatory muscle, the symptoms of eyelid spasm and perioral expression muscle twitch completely resolved after radiofrequency treatment of bilateral stylomastoid foramen facial nerve; for ODS, which involves only the masticatory muscle, bilateral foramen oval trigeminal mandibular branch radiofrequency could eliminate masticatory muscle spasm; and for B-ODS, which involves BDS combined with ODS, extracranial radiofrequency therapy of the mandibular branch of the facial nerve and trigeminal nerve was performed successively to eliminate BDS and masticatory muscle spasm, respectively. It is conceivable that if Meige’s syndrome affects more cranial muscles, the cranial nerves that transmit motor signals to the corresponding muscles will need to be treated with radiofrequency.

In contrast to facial spasm which is caused by irregular, involuntary, and painless clonus of unilateral facial muscles due to compression of the root of the facial nerve by accompanying blood vessels (Chaudhry et al., 2015), the cranial dystonia of Meige’s syndrome is often symmetrically distributed on both sides of the central axis of the face, and its pathogenesis is not caused by the accompanying vascular compression of the root of the facial nerve in the anterior pontine cistern. Therefore, Meige’s syndrome cannot be treated by craniotomy microvascular decompression (Pandey and Sharma, 2017).

In the current case series, we observed that after radiofrequency treatment of bilateral nerves, mild facial paralysis occurred simultaneously, there was no quarrel or skew, but the sucking ability significantly decreased, leaving the patient unable to complete the smiling action, and no other complications occurred. After radiofrequency surgery on the bilateral mandibular branches of the trigeminal nerve, there was no difficulty in opening the mouth. Nonetheless, hypoesthesia and numbness persisted in the innervation area of bilateral mandibular branches. However, two patients had recurrences at the 18th and 22nd months after 24 months of follow-up. No recurrence was reported in the other cases, which may be attributable to the short follow-up time in these cases. However, in our previous experience with extracranial radiofrequency treatment of trigeminal neuralgia, 11–13, 22–24 the effects lasted for 2–5 years.

## Conclusion

CT-guided extracranial radiofrequency ablation of the bilateral stylomastoid foramen facial nerve and/or foramen oval trigeminal mandibular branch may be a viable treatment option for Meige’s syndrome. Apart from botulinum toxin injection and deep brain stimulation, neuro-extracranial radio frequency technology may become a new treatment for Meige’s syndrome.

## Data availability statement

The original contributions presented in this study are included in the article/Supplementary material, further inquiries can be directed to the corresponding author.

## Ethics statement

This study was approved by the Ethics Committee of the Affiliated Hospital of Jiaxing University, Jiaxing, China (LS2019-013) on 20 May 2019. The patients/participants provided their written informed consent to participate in this study. Written informed consent was obtained from the individual(s) for the publication of any potentially identifiable images or data included in this article.

## Author contributions

BH was responsible for the project conception and patients’ interviews. X-DD was responsible for the literature search. MY was responsible for the project conception. W-HY was responsible for the video. Q-HZ was responsible for the project conception and manuscript review. All authors contributed to the article and approved the submitted version.

## Supplementary material

Supplementary Video 1 Facial muscles beat rhythmically with facial nerve stimulation.

Supplementary Video 2 The mandibular joint beats rhythmically when the mandibular branch of the trigeminal nerve is stimulated.

## Abbreviations

BDS: blepharospasm dystonia syndrome
ODS: oromandibular dystonia syndrome
B-ODS: blepharospasm combined with oromandibular dystonia syndrome.
